# Small molecule inhibitors of mesotrypsin from a structure-based docking screen

**DOI:** 10.1371/journal.pone.0176694

**Published:** 2017-05-02

**Authors:** Olumide Kayode, Zunnan Huang, Alexei S. Soares, Thomas R. Caulfield, Zigang Dong, Ann M. Bode, Evette S. Radisky

**Affiliations:** 1 Department of Cancer Biology, Mayo Clinic Comprehensive Cancer Center, Jacksonville, Florida, United States of America; 2 The Hormel Institute, University of Minnesota, Austin, Minnesota, United States of America; 3 Photon Sciences Directorate, Brookhaven National Laboratory, Upton, New York, United States of America; 4 Department of Neuroscience, Mayo Clinic College of Medicine, Jacksonville, Florida, United States of America; Stanford University, UNITED STATES

## Abstract

*PRSS3*/mesotrypsin is an atypical isoform of trypsin, the upregulation of which has been implicated in promoting tumor progression. To date there are no mesotrypsin-selective pharmacological inhibitors which could serve as tools for deciphering the pathological role of this enzyme, and could potentially form the basis for novel therapeutic strategies targeting mesotrypsin. A virtual screen of the Natural Product Database (NPD) and Food and Drug Administration (FDA) approved Drug Database was conducted by high-throughput molecular docking utilizing crystal structures of mesotrypsin. Twelve high-scoring compounds were selected for testing based on lowest free energy docking scores, interaction with key mesotrypsin active site residues, and commercial availability. Diminazene (CID22956468), along with two similar compounds presenting the bis-benzamidine substructure, was validated as a competitive inhibitor of mesotrypsin and other human trypsin isoforms. Diminazene is the most potent small molecule inhibitor of mesotrypsin reported to date with an inhibitory constant (*K*_i_) of 3.6±0.3 μM. Diminazene was subsequently co-crystalized with mesotrypsin and the crystal structure was solved and refined to 1.25 Å resolution. This high resolution crystal structure can now offer a foundation for structure-guided efforts to develop novel and potentially more selective mesotrypsin inhibitors based on similar molecular substructures.

## Introduction

*PRSS3*/Mesotrypsin is one of three tryptic digestive enzymes produced and secreted from the human pancreas. Unlike other trypsin isoforms, it possesses resistance to proteinaceous trypsin inhibitors [1–3]. Additionally, mesotrypsin displays differential activity from the other two human trypsin isoforms, displaying reduced capability for activating pancreatic zymogens [3], reduced activation of protease activated receptors (PARs) [4, 5], and elevated proteolytic activity towards proteinaceous trypsin inhibitors [3, 6–10]. Mesotrypsin represents a minor constituent of pancreatic secretions, and is hypothesized to be important in the digestion of trypsin inhibitors from the diet [3]. *PRSS3* expression outside of the digestive tract has also been described; for example, splice isoform gene products of *PRSS3* known as trypsinogen 4 and 5 are found in the brain and epidermis [10–12]. Tissue specific expression of these isoforms is driven from distinct promoters, while the active forms of these proteases are of identical sequence to pancreatic mesotrypsin. In the skin, mesotrypsin has been found to contribute to epidermal barrier permeability formation, keratinocyte terminal differentiation, and epidermal desquamation [12–14] while no function has yet been attributed to the high levels expressed in the brain.

Mesotrypsin expression has been observed in a number of different cancers including non-small cell lung cancer (NSCLC) [15], breast [16], esophageal [17], pancreatic [18], prostate [19], and ovarian cancers [20]. Elevated *PRSS3*/mesotrypsin expression is associated with poor prognosis in NSCLC, pancreatic, prostate, and ovarian cancers [15, 18–20]. In prostate cancer this overexpression is associated with a metastatic phenotype, which is recapitulated in culture models and mouse xenograft studies [19]. Studies with cell and animal models have likewise functionally implicated mesotrypsin as a contributor to progression and metastasis of pancreatic cancer [18]. Upregulation of mesotrypsin in endothelial cells is associated with cell migration and angiogenic stimulation by degradation of tissue factor pathway inhibitor-2 (TFPI-2) [9]. This and other data taken together advocate that targeting mesotrypsin may represent an effective therapeutic strategy for intervening in cancer progression and metastasis.

Trypsin family members share high sequence identity and homology making selective targeting a challenge. Our group has previously employed structure-based and directed evolution protein engineering efforts focused on modifying natural serine protease inhibitors to identify stabilizing mutations which would confer improved affinity towards mesotrypsin while also slowing the rate of proteolysis [21–23]. Another study used the phage display technique to identify small peptide inhibitors of mesotrypsin [24]. However, peptide and protein-based therapeutics face difficulties during drug development due to challenges including poor cellular uptake, poor solubility and pharmacokinetics, and issues relating to immunogenicity, storage, stability, and formulation [25]. These are areas in which small molecule therapeutics can offer advantages over peptide or protein-based drugs. Efforts to develop mesotrypsin small molecule inhibitors have been limited to one study by Karle and associates [26], in which the compounds had high IC_50_ values (upper micromolar to millimolar range), potentially limiting their utility.

To identify better small molecule inhibitors, here we describe a structure guided effort utilizing virtual screening to identify compounds with predicted activity towards mesotrypsin. Three compounds displaying inhibitory activity towards mesotrypsin were identified, and evaluated for selectivity among human trypsin isoforms. Additionally, we report a 1.25 Å crystal structure of the top candidate in complex with mesotrypsin, which we anticipate can serve as a springboard for further future development of mesotrypsin selective compounds.

## Results and discussion

### Molecular docking identifies candidate molecules with prospective mesotrypsin inhibitory activity

An ensemble docking-based virtual screening [27] of compounds from the FDA and NPD databases was conducted using three crystal structures of mesotrypsin (PDB IDs: 3P92 [22], 3P95 [22], and 1H4W [2]). Positive hits were chosen based on low docking scores to mesotrypsin and crucial hydrogen bond interactions observed with mesotrypsin binding pocket residues such as Asp-189, Ser-190, Gln-192, Arg-193, and Gly-216 [22]. The docking scores were formulated on the “extra-precision” XP Glide software algorithm which considers factors such as: lipophilicity, displacement of water, hydrogen bonding and electrostatic interactions, and metal ion/ligand interactions as favorable interactions, while the desolvation of polar or charged groups, restriction of motion, and the entropic cost of binding adversely affect docking score [28, 29]. Twenty-eight top compounds were computationally predicted to exhibit activity towards mesotrypsin based on the virtual screening; details of selection criteria are described in Materials and Methods.

### Virtual screening and experimental validation identify bis-benzamidine compounds with mesotrypsin inhibitory activity

**Fig 1:**
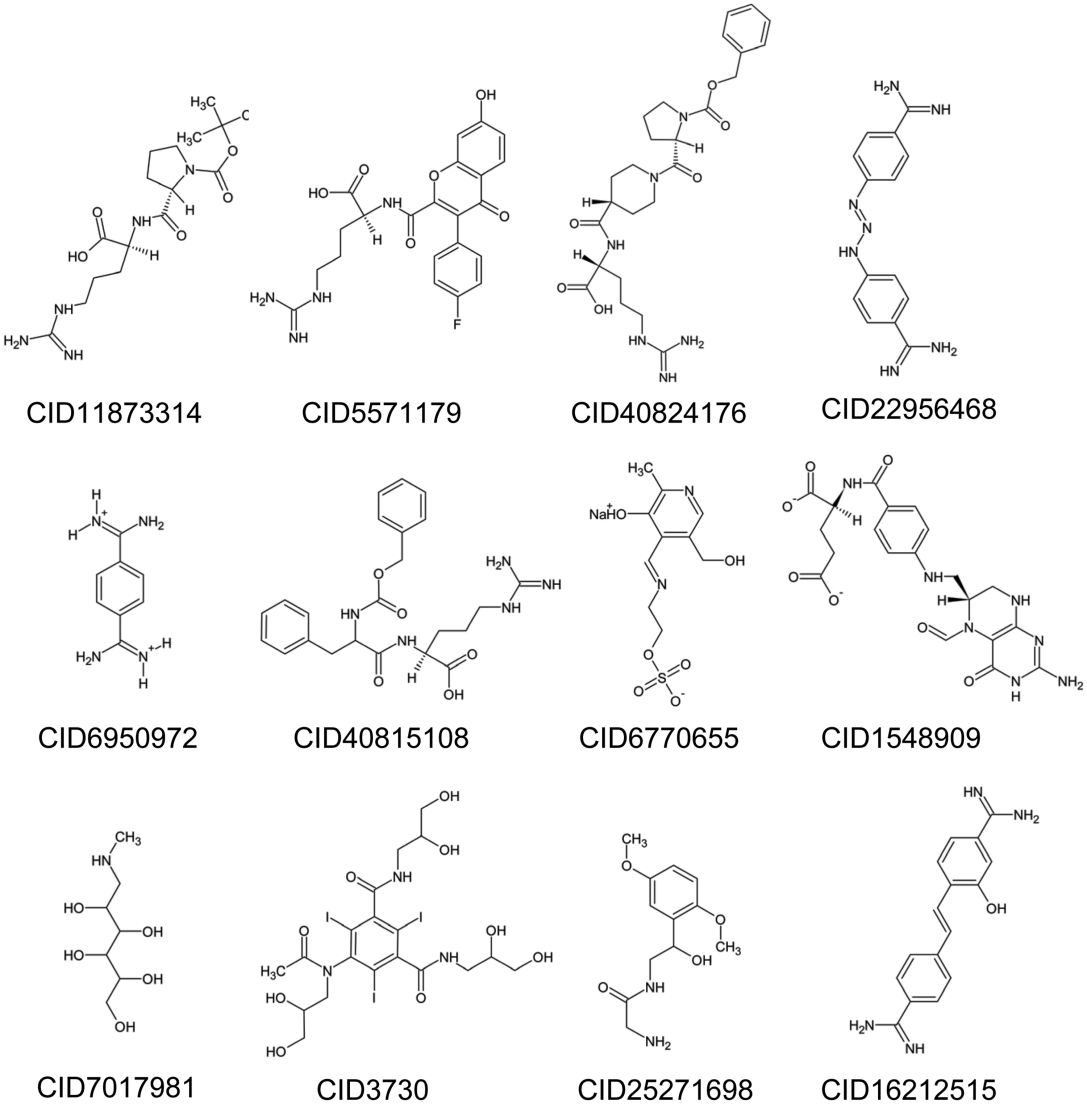
Commercially available compounds from virtual screen of FDA and NPD databases.

**Fig 2:**
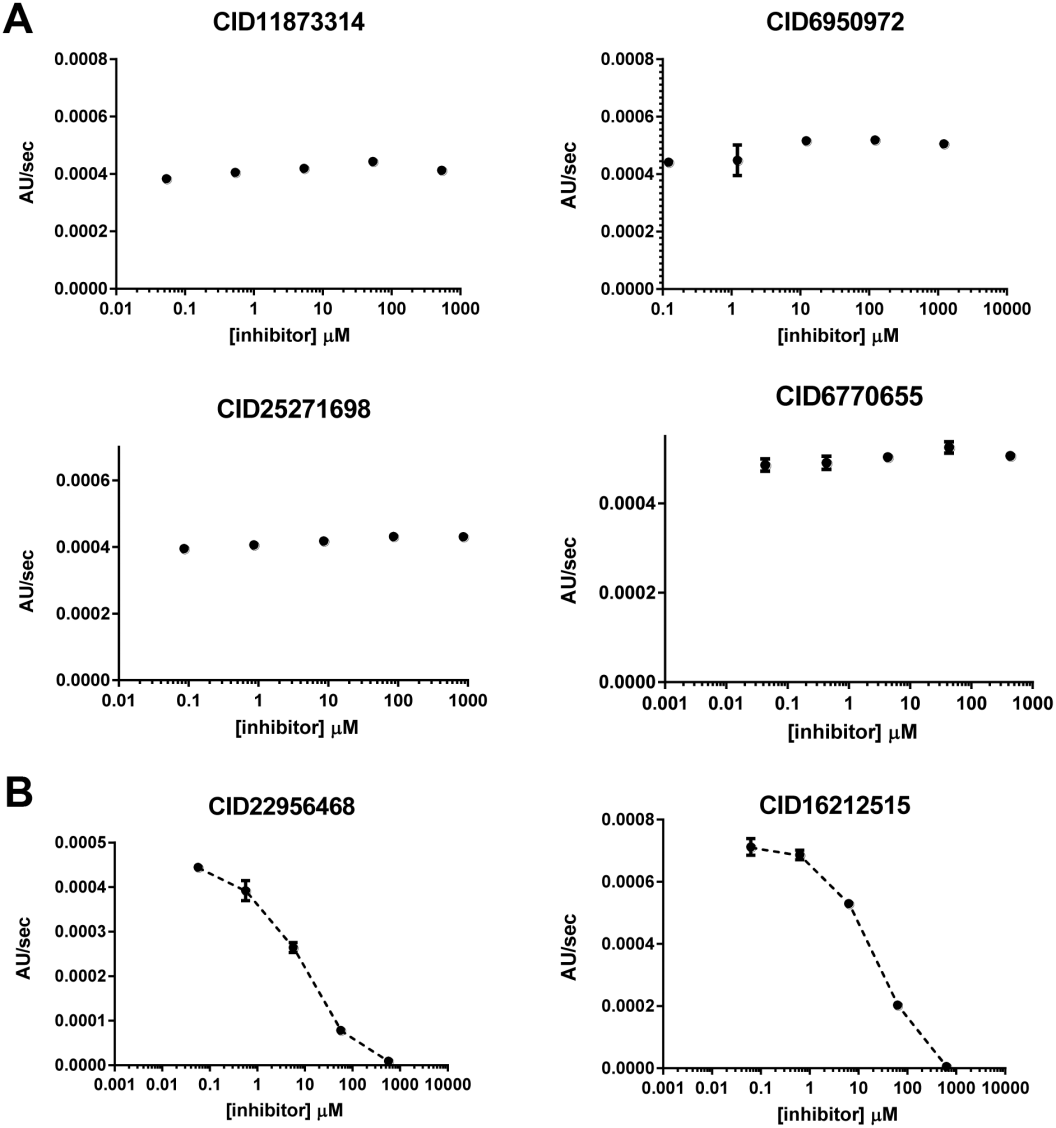
Mesotrypsin inhibitory activity of select compounds from virtual screening effort. A) Log-Linear plots from the activity assay for representative compounds that showed no activity towards mesotrypsin. B) Log-Linear plots of mesotrypsin inhibition by compounds CID22956468 and CID16212515, subsequently identified as diminazene and hydroxystilbamidine, respectively.

From the twenty-eight top compounds identified in the virtual screen, twelve compounds were found to be readily commercially available and were therefore procured and evaluated for inhibitory activity towards mesotrypsin (Fig 1, S1 Table). Compounds were tested by an *in vitro* activity assay utilizing the colorimetric peptide reporter substrate N-Cbz-Gly-Pro-Arg-pNA. The assay monitors the rate of para-nitroaniline production after substrate cleavage by mesotrypsin. Of the twelve compounds tested, ten displayed no inhibitory activity towards mesotrypsin into the low millimolar range. Conversely, two compounds, diminazene (CID 22956468) and hydroxystilbamidine (CID 16212515) exhibited inhibitory activity towards mesotrypsin in the low micromolar range (Fig 2A and 2B).

**Fig 3:**
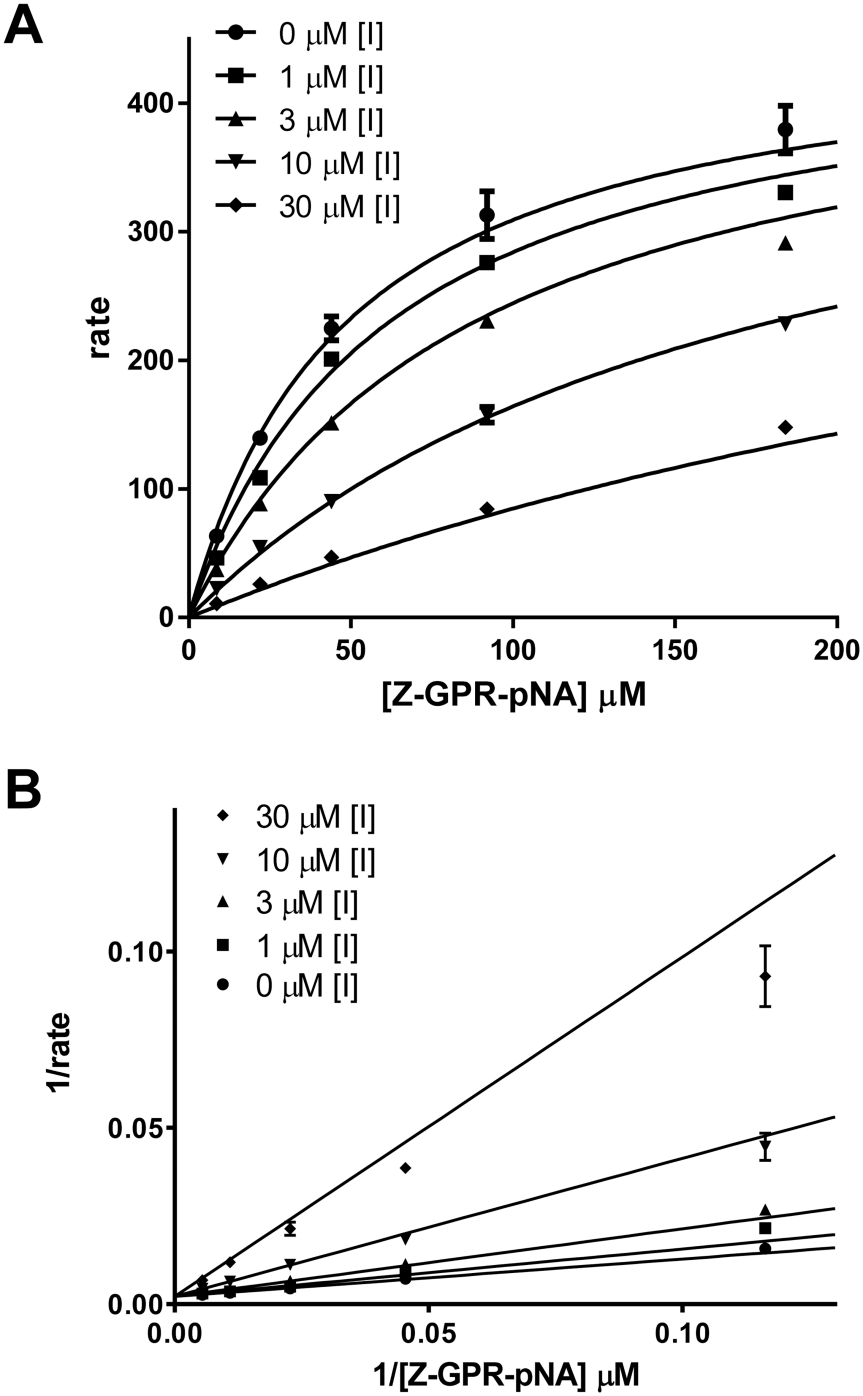
Diminazene (CID 22956468) is a competitive inhibitor of mesotrypsin. A) Global fit for the competitive inhibition equation to inhibition data and B) Lineweaver-Burk transform reveal competitive mode of inhibition for diminazene as evidenced by convergence of the Lineweaver-Burk plot on the y-axis. Results are representative of duplicate independent experiments.

**Fig 4:**
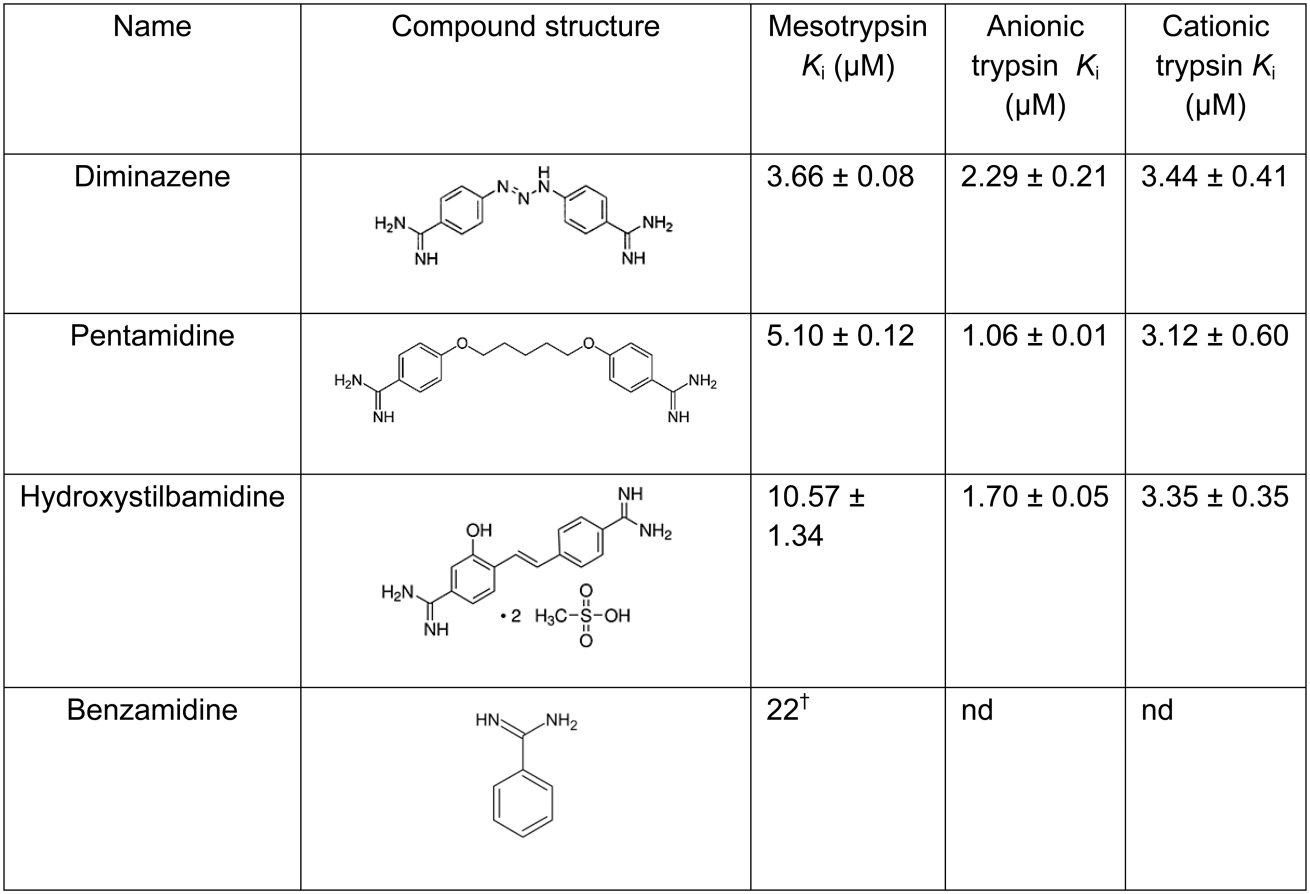
Structural comparison and selectivity of validated mesotrypsin inhibitors. ^†^Benzamidine inhibition constant versus trypsin IV (mesotrypsin) is as reported in [2].

The two inhibitory compounds are structurally similar bis-benzamidine analogs differing in the composition of the linker region. Although our virtual screen had not employed any prior knowledge about trypsin inhibitory compounds, a literature search of these compounds revealed that diminazene and pentamidine, another bis-benzamidine analog, are both trypanocidal drugs with known inhibitory activity towards trypanosome serine oligopeptidase [30], while also displaying activity against bovine trypsin [30, 31]. Upon obtaining pentamidine, we assayed the three bis-benzamidine compounds against mesotrypsin over multiple substrate and compound concentrations to determine the inhibition mode: competitive, non-competitive, or mixed inhibition. All three compounds exhibited a competitive inhibition mode of binding toward mesotrypsin (shown for diminazene in Fig 3), with inhibition constants (*K*_i_) of 3.66, 5.10, and 10.57 μM for diminazene, pentamidine, and hydroxystilbamidine respectively (Fig 4).

To assess compound selectivity we determined *K*_i_ for the three compounds towards related human trypsin isoforms trypsin 1 (cationic trypsin) and trypsin 2 (anionic trypsin). All three compounds displayed the tightest affinity for anionic trypsin, slightly reduced affinity for cationic trypsin, but presented marginal selectivity among these two isoforms. Diminazene bound to mesotrypsin most tightly while also being the least selective for the other trypsin isoforms (Fig 4), and was in turn selected as the best candidate compound for further investigation based on this profile.

### High resolution crystal structure of diminazene in complex with mesotrypsin

We set out to crystallize diminazene with mesotrypsin to gain a structural view of this candidate compound bound within the mesotrypsin active site. Protein crystals were grown at room temperature, harvested, and cryoprotected prior to data collection. Data were collected from one crystal which diffracted to 1.25 Å resolution. The diminazene-mesotrypsin complex crystallized in the P2_1_2_1_2_1_ space group having the unit cell dimensions of a = 40.92, b = 64.44, c = 80.68 and αβγ = 90° (Table 1). The structure was solved using molecular replacement.

**Table 1:** X-ray data collection and crystal refinement statistics.

mesotrypsin-diminazene	
PDB code	5TP0
*Data collection*	
Space group	P2_1_2_1_2_1_
Cell dimensions	
*a*, *b*, *c* (Å)	40.92, 64.44, 80.68
α, β, γ (°)	90, 90, 90
Resolution (Å)	50–1.25 (1.27–1.25)
R_merge_	0.045 (0.222)
R_meas_	0.047 (0.250)
R_p.i.m_	0.013 (0.112)
CC1/2	ND (0.954)
I/σI	52.1 (5.1)
Completeness (%)	98.4 (82.3)
Redundancy	11.1 (4.5)
*Refinement*	
Resolution (Å)	50–1.25
No. reflections	55799
R_work_ / R_free_	11.8 / 13.7
No. atoms	
Protein	1772
Ligand/ion	42 (1 CA, 4 SO4, 1 DRG)
Water	226
B-factors	
Protein	12
Ligand/ion	18
Water	27
Ramachandran statistics	
Favored (%)	99
Allowed (%)	1
Outliers (%)	0
R.m.s deviations	
Bond lengths (Å)	0.0252
Bond angles (°)	2.17

Values from highest resolution shell are shown in parentheses.

ND, CC1/2 for full dataset was not reported by HKL-2000.

**Fig 5:**
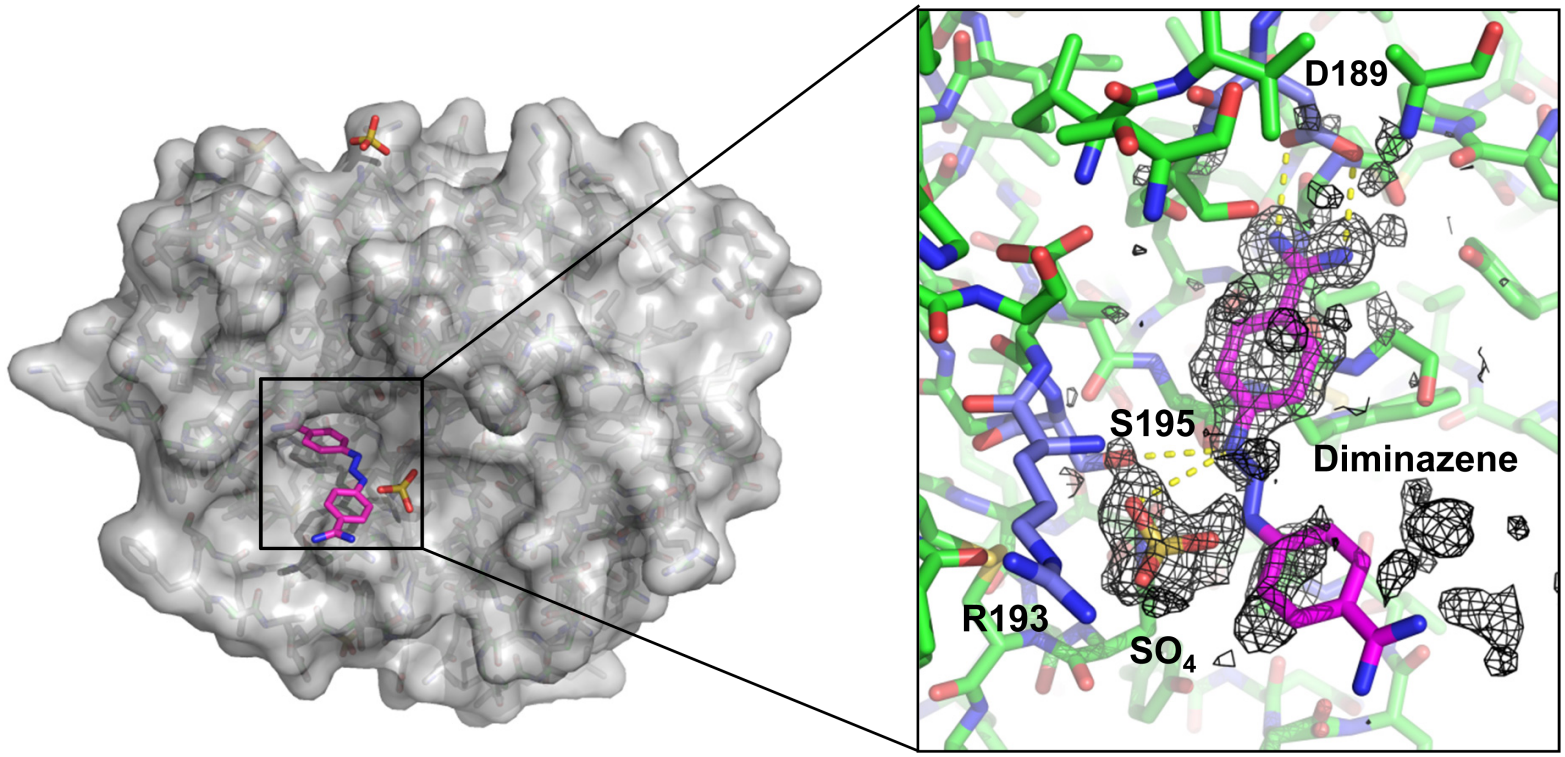
Crystal structure of mesotrypsin in complex with diminazene. Mesotrypsin Arg-193 is shown with carbon atoms in periwinkle, other mesotrypsin residue carbons are in green, and diminazene is shown with carbon atoms in magenta. Omit difference map contoured at 2.0σ shown in the right panel displays well-defined electron density for the upper benzamidine group bound within the specificity pocket. The remainder of the molecule, unmodelled in the deposited coordinate file, is disordered as portrayed by the diffuse density around the lower benzamidine moiety, revealing that the drug is able to adopt multiple conformations within the solvent channel.

**Fig 6:**
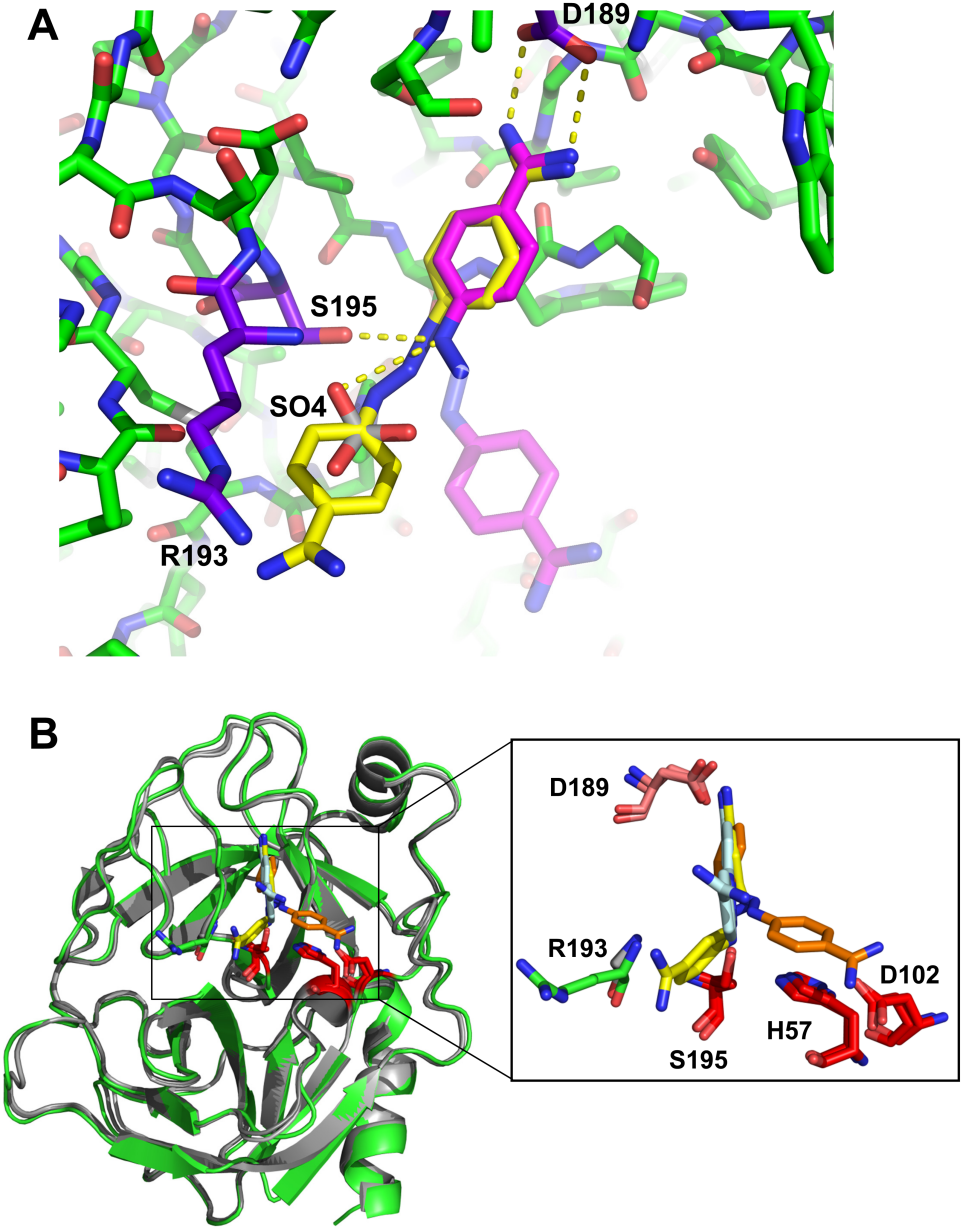
Conformational isomers of diminazene within the trypsin active site. **A)** Structural overlay of the top diminazene docking pose (yellow) and crystallographic structure (Mesotrypsin, green; Arg-193, purple; diminazene, magenta) show good correlation between the predicted and observed ligand positioning in the mesotrypsin specificity pocket, with a ligand RMSD of 0.614 Å. Atoms of the second benzamidine moiety lacking defined density in the crystal structure are represented as transparent sticks, and were not used in the RMSD calculation. **B)** A structural overlay of the mesotrypsin-diminazene docking pose (mesotrypsin, green; diminazene, yellow) with bovine trypsin (gray) in complex with diminazene (orange, cyan) from entries 3GY2 and 3GY6 [31] highlights the multiple diminazene binding conformations possible within the trypsin active site. Catalytic triad residues are shown in red, specificity pocket residue Asp-189 in salmon, and mesotrypsin Arg-193 shown in green.

Following refinement, well defined density was observed for the first benzamidine moiety of diminazene, which was observed to bind within the specificity pocket of mesotrypsin (Fig 5). Trypsins cleave after basic residues lysine and arginine, and use the charge complementarity of the specificity pocket to recognize their substrates. This interaction and specificity is mediated by Asp-189 which lies at the base of the pocket and forms salt bridge interactions with the ε-amino or guanidinium group of the substrate residue. In the present structure, nitrogen atoms NAA and NAC of diminazene display hydrogen bonding contacts with Asp-189 of the specificity pocket. The positioning of diminazene in the specificity pocket closely recapitulates the position predicted for the ligand by the docking screen, with a ligand RMSD of 0.614 Å (Fig 6A). The second benzamidine moiety of the diminazene molecule exhibited diffuse density, which may be attributable to exposure to the solvent channel, permitting multiple ligand conformations (Fig 5). We anticipate the conformational disorder observed in diminazene could arise in part from cis-to-trans isomerization of the triazene linker [32, 33]. Diminazene has been previously observed as two distinct conformational isomers bound to bovine trypsin in PDB IDs: 3GY2, 3GY6 (Fig 6B) [31].

### Molecular docking predicts potential diminazene interaction with distinctive mesotrypsin Arg-193 residue

Mesotrypsin differs from other human trypsin family members at twenty-eight residues; Arg-193 is one of the most critical because it is located within the active site, at a position where almost all other trypsin and chymotrypsin family serine protease members possess a highly conserved glycine residue. This critical residue is a major contributor to mesotrypsin’s resistance to inhibition and enhanced proteolytic activity towards protein protease inhibitors [2, 3, 6, 34]. Although our high resolution crystal structure revealed the second benzamidine ring of diminazene to be poorly ordered within the mesotrypsin active site, the top ranking binding pose of this molecule from the virtual screen presented a conformational isomer with diminazene interaction at Arg-193 (Fig 6). We hypothesize that future compound optimizations may enhance the interaction with this distinctive active site residue, producing small molecule inhibitors with improved selectivity toward mesotrypsin versus other trypsins and related proteases. Derivatization efforts may include rigidifying the compound scaffold, maximizing stability of conformers that contact Arg-193, and evaluating substitutions of the amidine moiety to optimize polar and electrostatic interactions at the Arg-193 guanidinium moiety. Scaffold “rigidification” is a known approach to improve potency and specificity of compounds toward macromolecular targets [35–38], which given the conformational disorder found in the diminazene co-crystal structure with mesotrypsin, is likely beneficial.

## Conclusions

Mesotrypsin has been identified as a facilitator of cancer progression thereby suggesting mesotrypsin could represent a druggable target, but currently no small molecule inhibitors demonstrate enough discrimination between trypsin family members. Mesotrypsin displays unique active site features and conformational dynamics [34] which can in turn influence substrate motions [39]. Diminazene, a bis-benzamidine analog with mesotrypsin inhibitory activity, presents an attractive starting point for the development of more selective mesotrypsin inhibitors. This new high resolution crystal structure of mesotrypsin complexed with diminazene could help facilitate derivatization efforts, addressing current pan-inhibition of human trypsins through rigidification of diminazene to select for a conformation that maximizes interactions with the non-conserved Arg-193 residue.

## Materials and methods

### Virtual screening using Glide

The crystal structures of mesotrypsin (PDB IDs: 3P92 [22], 3P95 [22], and 1H4W [2]) were downloaded from the Protein Data Bank [40]. The three mesotrypsin structures were further converted into all-atom, fully prepared receptor model structures by using the Protein Preparation Wizard module in Maestro (Maestro version 9.2, Schrödinger, LLC). Default settings were used, except that bovine pancreatic trypsin inhibitor (BPTI) variants were cut to leave only the Arg-15 ligand residue in 3P92 and 3P95 structures. Next, the docking receptor grid was created using the Receptor Grid Generation feature (Glide version 5.7, Schrödinger, LLC). The grid boxes and centers were generated using default settings by identifying the mesotrypsin ligands BPTI Arg-15 (for 3P92 and 3P95) and BEN (for 1H4W). Meanwhile, the FDA and NPD Databases were downloaded from the ZINC database (http://zinc11.docking.org/vendor0/) and further processed by using Schrodinger's LigPrep (LigPrep, version 2.5, Schrödinger, LLC).

We used the Virtual Screening Workflow (VSW) in Schrödinger to run the docking screening of FDA and NPD databases targeted to an ensemble of three receptor grids (3P92, 3P95 and 1H4W). In the VSW procedure, the docking of the compounds in a database into each receptor grid was hierarchically performed using the Glide program (Glide version 5.7, Schrödinger, LLC) through three accuracy levels: HTVS, SP (Standard Precision) [28] and XP (Extra Precision)-docking [29] in successive steps. The screening processes outputted six rank lists of compound leads, each list corresponding to the screening of one database to one receptor grid. Docked poses of all compounds with docking scores <-10.0 kcal/mol versus one or more receptor grid using the XP algorithm were visually examined for hydrogen bond interactions observed with mesotrypsin binding pocket residues including Asp-189, Ser-190, Gln-192, Arg-193, and Gly-216. Twenty-eight of the compounds showed four or more direct hydrogen bonds with the enzyme, and thus were selected for a final list representing all of the hits with XP docking scores lower than -10 kcal/mol versus one or more of the three receptor grids, and also possessing four or more hydrogen bonds with mesotrypsin. Among these 28 compounds, 12 were found to be readily commercially available and thus were procured for experimental validation.

### Protein expression and purification

Recombinant human anionic trypsinogen, human cationic trypsinogen and human mesotrypsinogen were expressed in *E*. *coli*, extracted from inclusion bodies, refolded, purified and activated with bovine enteropeptidase as described in previous work [6, 22].

### Compounds

Milligram quantities of each compound were requested or procured from commercial sources. Diminazene (CID 22956468), pentamidine (CID 4735), hydroxystilbamidine (CID 16212515), and CID 6950972 were received from the National Cancer Institute Developmental Therapeutics Program (NCI-DTP) (Rockville, MD). CID 3730 and 25271698 were purchased from Prestwick Chemical (San Diego, CA), and CIDs 11873314, 5571179, 40824176, 40815108, 6770655, 1548909, 7017981 were purchased from InterBioScreen Ltd (Russia)

### Mesotrypsin activity and competitive inhibition assays

Active mesotrypsin was quantified by active site titration as described in [21] using 4-nitrophenyl-4-guanidinobenzoate (NPGB) from Sigma. Concentrations of colorimetric peptide reporter substrate N-Cbz-Gly-Pro-Arg-pNA (Sigma) for the activity and competitive inhibition assay were determined by end point assay. Working stocks for compounds were diluted in dimethylsulfoxide (DMSO) and stored at -20°C. Compounds were assessed for mesotrypsin inhibitory activity in reactions carried out in cuvettes at 37°C in a Varian Cary-100 spectrophotometer. Reactions consisted of 0.25 nM enzyme, 100 μM reporter substrate, 100 mM Tris-HCl pH 8.0, 1 mM CaCl_2_, and a compound concentration range between 50 nM to 500 μM in the assay. Reactions were followed for approximately three minutes, with the initial rates determined by the linear increase in absorbance resulting from para-nitroaniline production (ε410 = 8480 M^-1^ cm^-1^). Absorbance versus compound concentration was plotted on a Linear-Log scale to determine IC_50_.

The competitive inhibition assay reactions consisted of 0.25 nM enzyme, five reporter substrate concentrations between 10 μM-200 μM, and five inhibitory compound concentrations between 1–30 μM. Reactions were followed for approximately 3 minutes, and the initial rate data were globally fitted using Prism (GraphPad Software, San Diego CA) by non-linear regression based on Eq 1. Results are from duplicate independent experiments.

$$\text{ V}=\;\frac{{\text{K}}_{\text{cat}}{\left[\text{E}\right]}_{\text{o}}\left[\text{S}\right]}{{\text{K}}_{\text{m}}\;\left(1+\frac{\left[\text{I}\right]}{{\text{K}}_{\text{i}}}\right)+\left[S\right]}$$ (1)

### Protein crystallization

Mesotrypsin was mixed with 1 mM diminazene at a total protein concentration of 4 mg/mL. Protein complex solution was mixed 1:1 (v/v) with reservoir solution, and crystallized via the hanging drop method over a reservoir solution containing 2 M ammonium sulfate, 0.1 M HEPES pH 7.5, and 2% PEG-400 at room temperature. Crystals were harvested, cryoprotected, and flash cooled in liquid N_2_. X-ray diffraction data were collected at beamline X25 at the National Synchrotron Light Source, Brookhaven National Laboratory. Data were collected at 100 K from one crystal that diffracted to 1.25 Å. Data were merged and scaled with HKL-2000 [41]. The crystal belonged to the space group P2_1_2_1_2_1_, with unit cell dimensions a = 40.92, b = 64.44, c = 80.68, αβγ = 90°, and contained 1 copy of the complex in the asymmetric unit. The structure was solved by molecular replacement using MOLREP in CCP4 [42], using as the search model the complex of benzamidine with mesotrypsin (also known as brain trypsin IV; PDB ID 1H4W). Refinement employed alternating cycles of manual rebuilding in WinCOOT [43, 44] and automated refinement using REFMAC5 [45]. The final stage of refinement included addition of solvent molecules into peaks greater than 1σ and within acceptable hydrogen bonding distance from neighboring protein atoms. The quality of the final models was analyzed using wwPDB validation tools [46]. The coordinates and structure factors have been submitted to the Worldwide Protein Data Bank under the accession code 5TP0. Structure figures were generated using PyMOL version 1.7.4 (Schrodinger, LLC).

## Supporting information

S1 Table Structures and docking scores of 12 compounds tested from the FDA and NPD databases. (PDF)
