# Supplementary material for: Small molecule inhibitors of mesotrypsin from a structure-based docking screen
Source: PLoS One. 2017 May 2;12(5):e0176694. doi: 10.1371/journal.pone.0176694 (PMC5413004; doi:10.1371/journal.pone.0176694)
Supplement: S1 Table — (PDF) [file pone.0176694.s001.pdf]

S1 Table: Structures and docking scores 12 compounds tested from the FDA and NPD databases.

ID	Structure	Docking score
CID11873314	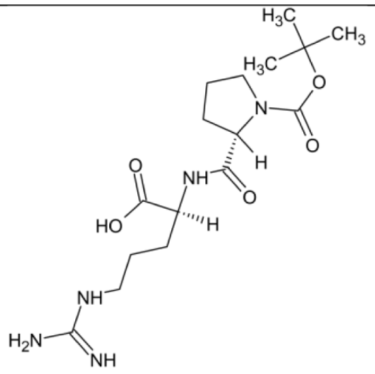	3P92: -11.193<br>3P95: -11.146
CID5571179	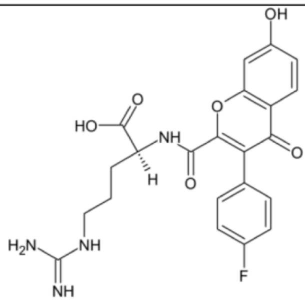	3P92: -11.087<br>3P95: -11.554
CID40824176	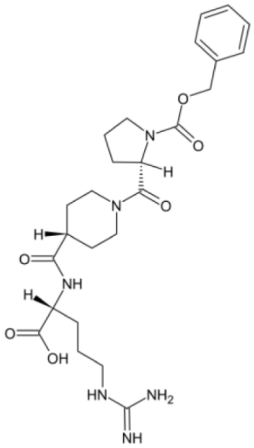	3P92: -10.992<br>3P95: -9.596<br>1H4W: -10.114
CID40815108	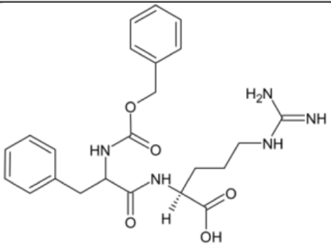	3P92: -10.863<br>3P95: -10.636

CID6950972	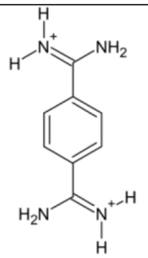	3P92: -10.548<br>3P95: -10.226<br>1H4W: -10.142
CID6770655	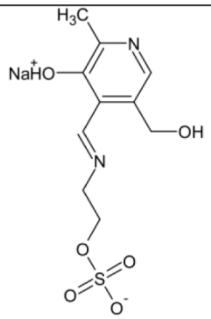	3P92: -10.248<br>3P95: -11.31<br>1H4W: -7.357
CID1548909	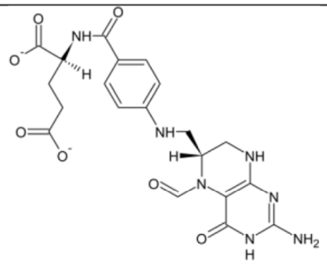	3P92: -11.832<br>3P95: -11.751<br>1H4W: -10.859
CID7017981	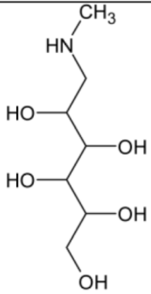	3P92: -11.747<br>3P95: -11.644<br>1H4W: -11.690
CID22956468	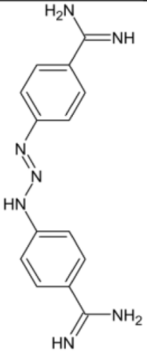	3P92: -10.847<br>3P95: -11.058<br>1H4W: -10.605

CID3730	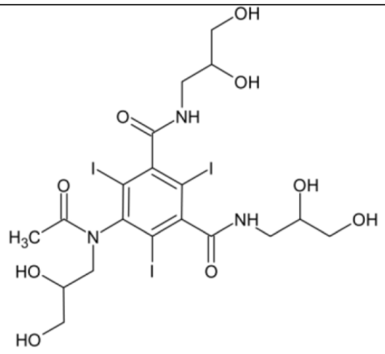	3P92: -11.291<br>3P95: -10.624<br>1H4W: -9.82
CID25271698	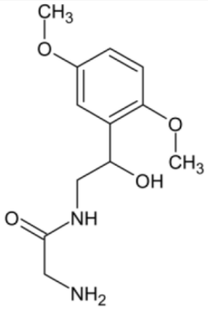	3P92: -10.585<br>3P95: -10.214<br>1H4W: -9.979
CID16212515	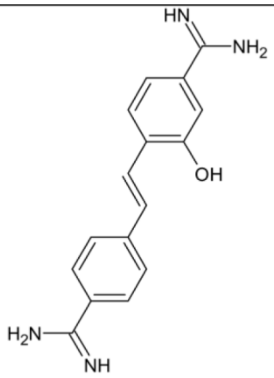	3P92: -11.472<br>3P95: -11.591<br>1H4W: -12.128
